# Comparison of Different Risk Perception Measures in Predicting Seasonal Influenza Vaccination among Healthy Chinese Adults in Hong Kong: A Prospective Longitudinal Study

**DOI:** 10.1371/journal.pone.0068019

**Published:** 2013-07-19

**Authors:** Qiuyan Liao, Wing Sze Wong, Richard Fielding

**Affiliations:** 1 Health Behaviors Research Group, School of Public Health, The University of Hong Kong, Hong Kong, China; 2 Department of Psychological Studies, The Hong Kong Institute of Education, Hong Kong, China; Fudan University, China

## Abstract

**Background:**

Risk perception is a reported predictor of vaccination uptake, but which measures of risk perception best predict influenza vaccination uptake remain unclear.

**Methodology:**

During the main influenza seasons (between January and March) of 2009 (Wave 1) and 2010 (Wave 2),505 Chinese students and employees from a Hong Kong university completed an online survey. Multivariate logistic regression models were conducted to assess how well different risk perceptions measures in Wave 1 predicted vaccination uptake against seasonal influenza in Wave 2.

**Principal Findings:**

The results of the multivariate logistic regression models showed that feeling at risk (β = 0.25, p = 0.021) was the better predictor compared with probability judgment while probability judgment (β = 0.25, p = 0.029 ) was better than beliefs about risk in predicting subsequent influenza vaccination uptake. Beliefs about risk and feeling at risk seemed to predict the same aspect of subsequent vaccination uptake because their associations with vaccination uptake became insignificant when paired into the logistic regression model. Similarly, to compare the four scales for assessing probability judgment in predicting vaccination uptake, the 7-point verbal scale remained a significant and stronger predictor for vaccination uptake when paired with other three scales; the 6-point verbal scale was a significant and stronger predictor when paired with the percentage scale or the 2-point verbal scale; and the percentage scale was a significant and stronger predictor only when paired with the 2-point verbal scale.

**Conclusions/Significance:**

Beliefs about risk and feeling at risk are not well differentiated by Hong Kong Chinese people. Feeling at risk, an affective-cognitive dimension of risk perception predicts subsequent vaccination uptake better than do probability judgments. Among the four scales for assessing risk probability judgment, the 7-point verbal scale offered the best predictive power for subsequent vaccination uptake.

## Introduction

Seasonal influenza is a major cause of excess morbidity and mortality in Hong Kong [1]–[3] and worldwide [4], [5]. Annual vaccination against the circulating influenza strain is currently the most important intervention for reducing influenza-associated mortality and hospitalizations [6], [7]. However, vaccination coverage for both the recommended priority/high-risk groups and healthy adults remain lower than expected in many western countries [8], [9]. Vaccination coverage rates in Hong Kong were 49%, 31% and 28% for young children, community elderly and the general adults, respectively [10]–[12], being much lower than those reported in equivalent US populations. Improving uptake of seasonal influenza remains an important public health issue.

In Hong Kong, seasonal influenza incidence shows peaks around January-March and July-August [13]. Government messages regarding seasonal influenza vaccination are usually launched around October/November each year to encourage people to receive influenza vaccine before the main influenza season. The Hong Kong government generally recommends all individuals without contraindications to receive the vaccine for self-protection [14]. In particular, priority groups including elderly living in residential care homes, long-stay residents of institutions for the disabled, persons aged 50 years or above, persons with chronic medical conditions, healthcare workers, children aged 6 months to five years, pregnant women, poultry workers, pig farmers and pig-slaughtering industry personnel are strongly recommended to take the vaccination [14]. In Hong Kong the vaccination of these priority groups is subsidized and therefore is free or at very low cost to recipients while non-priority adults must pay the full (∼US$20–25) cost of the vaccination.

Since influenza vaccination remains optional in Hong Kong and many other countries, vaccination uptake depends primarily on individuals' assessments of their personal risk from influenza versus influenza vaccine [15]. Risk perception, defined as individual cognitive judgments about personal probability of encountering negative events, is a core component of cognitive health behavioral models such as the Health Belief Model (HBM) [16] and Protection Motivation Theory (PMT) [17]. Substantial research has linked heightened perceptions of disease risk with subsequent adoption of health behaviors including cancer screening, healthy lifestyle practices, adherence to medical treatment and prevention of infectious diseases [18]–[21]. However, study findings about the association between risk perception and adoption of health behaviors are inconsistent. Reported associations between risk perception and uptake of health behaviour ranged from large and significant to negligible and insignificant [18]–[20]. Methodological issues including variability in assessing and defining risk perception as well as in study designs and study populations potentially account for these inconsistent results [20]–[22]. For vaccination, reviews have suggested that better assessment of the association between risk perception and vaccination uptake could be obtained if risk perception is measured in the way that (1) perceived risk is conditional on “not getting vaccinated”; (2) the risk refers to personal risk rather than the general risk and (3) the risk of the time frame is clearly specified [20]. Sample question could be “*without taking vaccination*, do you think *your* chance of getting influenza *in the coming year* is...”. Studies also suggested that the association between risk perception and vaccination uptake is stronger in prospective studies than in cross-sectional studies [20].

Other than the above methodological issues, risk perception scales constructed from different conceptual groundings could vary in terms of strength of associations with preventive actions [23]. The first approach assumes that people are able to adequately conceptualize and express their probability estimates about encountering a negative event in a verbal or numeric way. In this paper, we use the term “probability judgment” [23] to refer to approaches asking participants to estimate the probability of their contracting influenza. The second approach, termed “belief about risk” [23], assumes that people have difficulties in judging the risk probability in terms of either verbal or numeric scales. Namely, an individual may have some general beliefs about whether his/her risk of contracting influenza is high or low but may not be able to clearly articulate how high or low. Both the first and second approaches define risk perception from a cognitive science perspective based on the (dubious) assumption that humans are rational and emotion-free in processing and acting on risk [24]. The third approach is termed “feeling at risk” attempts to captures both cognitive and affective components of risk perception has been proposed to be better in predicting behavioral change [23]. Weinstein et als' study [23] found that feeling at risk predicted influenza vaccine uptake better than did beliefs about risk and probability judgments. Another finding of Weinstein and others' study is that among items for assessing probability judgment, the one with seven-choice verbal scale was the best predictor for vaccination uptake [23]. Other studies suggest that risk perception measured with verbal scale was better in predicting behavioral change than that measured with numeric percentage scale [25], [26]. However, all these studies were conducted among western respondents whose native languages were predominantly English. It is unknown whether the findings remain applicable to Chinese populations when the risk perception measures are translated from English into Chinese. Chinese people comprise the largest ethnic group worldwide. Influenza is a global problem and the global distribution of significant Chinese communities calls for a better understanding of how Chinese people perceive influenza risk and its association with vaccination uptake. Therefore, this study translated the English version of different risk perception measures assessed in Weinstein and others' study [23] into Chinese and assessed these risk perception measures as predictors of influenza vaccination uptake among Chinese adults.

## Methods

### Ethics Statement

The study obtained ethics approval from the Institutional Review Board of the City University of Hong Kong. Since the study was an online survey, respondents read the consent online and checked on a box to indicate their consent for participating in the survey.

### Participants and procedures

This study was a two-wave longitudinal survey conducted during major influenza seasons in Hong Kong. The baseline data were collected in Jan-Mar 2009 and the follow-up data were collected in Jan-Mar 2010. During data collection periods, all students, faculty and staff from the City University of Hong Kong (CityU) were invited to participate in the study by emails containing a hyperlink to the web questionnaires. To improve response rate, target subjects were notified that the first 600 respondents who completed the baseline or the follow-up surveys would receive a food coupon valued at HK$20 (∼US$2.56). A reminder was sent out on weekly basis to target subjects who had not participated in the survey during the entire period of data collection. Data collection stopped at the end of March in each wave.

This survey principally aimed to investigate people's vaccination uptake against seasonal influenza and their related perceptions. However, the current survey was unexpectedly influenced by the occurrence of the 2009 influenza pandemic (A/H1N1). In Hong Kong, the A/H1N1 epidemic occurred between data collection of the two waves, starting to spread widely in the middle of June, peaking in September and petering out in Early November 2009 [27]. A/H1N1 vaccine was available for priority groups from late December 2009 and for the general public from late of January 2010 in Hong Kong [28]. The campaign of seasonal influenza vaccination overlapped with the campaign of A/H1N1 vaccination. Therefore, It is possible that some respondents may have received A/H1N1 vaccine by the time Wave 2 data were collected in Jan-Mar 2010.

### Study measures

A standardized questionnaire based on Weinstein et al.'s study [23] was used for data collection. Before being uploaded to the CityU intranet website, the survey questionnaire was translated from English into Cantonese and back-translated into English using standard ethnographic procedures to check the accuracy of translation and was pre-tested for acceptability, content validity and comprehensibility. The Chinese version of the questionnaire was used for data collection. Questionnaires for the surveys in Wave 1 and Wave 2 were similar except that 21 items on perceptions and vaccination related to A/H1N1 were included in Wave 2. Measures for this paper were described below.

#### Risk perception measures

According to Weinstein and others' study [23], risk perception measures were constructed in three different ways, corresponding to risk probability judgment, beliefs about risk and feeling at risk. Four items addressed risk probability judgment with different response scales (either verbal or numeric scales) including one 2-point verbal scale (unlikely/likely), one 6-point verbal scale (extremely likely/very likely/somewhat likely/somewhat unlikely/unlikely/very unlikely), one 7-point verbal scale (almost zero/very small/small/moderate/large/very large/almost certain) and one percentage numeric scale (ranged from 0% to 100%). Details of each item for measuring risk probability judgment and their response scales are presented in Table 1. Beliefs about risk were addressed by asking about respondents' agreement (agree strongly/agree mildly/disagree mildly/disagree strongly) on two belief statements including “without a flu shot, I'm sure I would get the flu next year” and “without a flu shot, I would expect to get the flu next year”. Finally, feeling at risk asked respondents for extent of agreement (agree strongly/agree mildly/disagree mildly/disagree strongly) with two feeling statements including “with no flu shot, I would feel that I'm going to get the flu next year” and “with no flu shot, I would feel very vulnerable to the flu next year” (Table 1). Each items for measuring risk perceptions was coded or recoded in the way that higher score indicates higher perceived personal risk of getting influenza. Although all these items were measured in Wave 1 and Wave 2 surveys, only the risk perception measures in Wave 1 were used for the analysis in this paper. Risk perception measures of Wave 2 will be used for other purposes for the study. Details about the risk perception measures and the mean as well as the standard deviation of each item were presented in Table****1.

**Table 1:** Summary of study measures for risk perception of seasonal influenza.

Measures	Items	Response scale	Mean (SD)
Risk probability judgment			
2-point verbal scale	If I don't get a flu shot, I think I am...	1 = unlikely to get the flu next year, 2 = likely to get the flu next year	0.54 (0.50)
6-point verbal scale	Without a flu shot, do you think you are likely to get the flu next year?	1 = extremely likely, 2 = very likely, 3 = somewhat likely, 4 = somewhat unlikely, 5 = unlikely, 6 = very unlikely	3.59 (1.05)
7-point verbal scale	If I don't get the flu shot, I think my chances of getting flu next year would be...	1 = almost zero, 2 = very small, 3 = small, 4 = moderate, 5 = large, 6 = very large, 7 = almost certain	3.59 (1.15)
Percentage scale	If I don't get the flu shot, I think my chances of getting flu next year would be...	0% = no chance, 5%, 10%, 20%, 30%, 40%, 50%, 60%, 70%, 80%, 90%, 100% = certain	4.66 (2.43)
Beliefs about risk			
Sure will get flu	Without a flu shot, I am sure I would get the flu next year.	1 = agree strongly, 2 = agree mildly, 3 = disagree mildly, 4 = disagree strongly	1.97 (0.67)
Expect to get flu	Without a flu shot, I would expect to get the flu next year.	1 = agree strongly, 2 = agree mildly, 3 = disagree mildly, 4 = disagree strongly	2.12 (0.67)
Feeling at risk			
Feel will get flu	With no flu shot, I would feel that I'm going to get the flu next year.	1 = agree strongly, 2 = agree mildly, 3 = disagree mildly, 4 = disagree strongly	2.11 (0.67)
Feel vulnerable to flu	With no flu shot, I would feel very vulnerable to the flu next year.	1 = agree strongly, 2 = agree mildly, 3 = disagree mildly, 4 = disagree strongly	2.13 (0.68)

#### Vaccination behaviours

Respondents were asked about whether they had ever been vaccinated against influenza or not (Yes/No) in Wave 1 survey (past influenza vaccination) and whether they received at least one dose of influenza vaccine during the preceding 12****months or not (Yes/No) in Wave 2 (vaccination uptake). In Wave 2, respondents were also asked whether they had received A/H1N1 vaccine or not (Yes/No).

Except for the above data, demographics including age, gender, education, marital status, occupation (student/employee) and related medical history such as diagnosis on chronic illness and whether having allergy to vaccine were also collected in Wave 1.

### Data analysis

Data analysis assessed how well different risk perception measures at Wave 1 predicted subsequent vaccination uptake between Wave 1 and Wave 2. First, to assess non-response bias, Pearson chi-square test compared demographics of respondents completing both Wave 1 and Wave 2 with those who completed Wave 1 but were lost in Wave 2. Univariate logistic regression was conducted to assess the associations between demographic factors and vaccination uptake. Demographics that were significantly associated with vaccination uptake were later used for adjustment in multivariate analyses. Spearman correlation coefficients were then computed to assess the relationships between different measures of risk perception and to assess the collinearity between different items for measuring risk perception. A high correlation (r>0.85) was taken to indicate that potential collinearity exists between two variables [29]. Spearman correlation coefficient was also calculated to assess the strength of correlation between each risk perception measure and vaccination uptake. Finally, A series of multivariate logistic regression models were run to compare the three different risk perception measures in predicting vaccination uptake. These multivariate logistic regression models served two purposes: (1) to assess the relative strengths of the four items that assessed risk probability judgment in predicting vaccination uptake and (2) to assess the relative strengths of risk probability judgment, beliefs about risk and feeling at risk in predicting vaccination uptake. To minimize multicollinearity, instead of entering all risk perception measures into a logistic regression model simultaneously, different pairings of risk perception measures (1+2; 1+3; 2+3) were separately enter into a logistic regression model. The relative strengths of different risk perception measures in predicting vaccination uptake could then be assessed by comparing the relative magnitudes of their corresponding standardized regression coefficients (β). By paring risk perception measures into a regression model, we could also assess whether different risk perception measures predicted different aspects of vaccination uptake. For example, two risk perception measures are likely predict the same aspect of vaccination uptake if they become insignificant in predicting vaccination uptake when paired in a regression model. However, if one risk perception measure remains significant while the other becomes insignificant in predicting vaccination uptake, then the significant measure possibly explains additional variance in vaccination uptake that is not explained by the other risk perception measure in the pair. All multivariate logistic regression models were adjusted by demographics significant in preliminary bi-variable analyses and all p values <0.05 were considered statistically significant. All statistics were conducted using STATA software (version 10.1; STATA Corp., College Station, TX). Raw data from the study are available on request to allow for reproducible analyses.

## Results

### Participants

By the end of Wave 1 data collection , a total of 1764 respondents (∼6% of the 28,728 employees and students of CityU) completed the questionnaire. A total of 1239 respondents were lost to follow-up in Wave 2, leaving only 525 (30%, 525/1761) respondents completing both Waves 1 and 2. Characteristics of respondents who completed both waves of the survey and those who were lost to follow-up in the second wave did not differ significantly except that respondents who were lost to follow-up were slightly younger (Table S1). Of the 525 respondents who completed both waves of the survey, 18 respondents who reported having had been vaccinated against A/H1N1 in Wave 2 and an additional two respondents who reported had ever been allergic to influenza vaccine were excluded, leaving 505 subjects for subsequent data analysis.

### Demographics and vaccination uptake

Of the 505 respondents in Wave 2, 57 (11%) reported having had received influenza vaccine over the past 12****months. Vaccination uptake (vaccination between Wave1 and Wave 2) did not significantly differ by gender and education obtainment of the respondents (Table****2). However, respondents who were older (aged ≥35****years: OR  = 3.24, 95% CI: 1.77–5.94), married or formerly married (OR  = 2.71, 95% CI: 1.56–4.72), employee (vs. student) (OR  = 2.68, 95% CI: 1.62–4.45), reporting a chronic condition (OR  = 4.55, 95% CI: 2.01–10.30) and having had received influenza vaccine in the past (OR  = 5.18, 95% CI: 2.84–9.45) were more likely to report vaccination uptake (Table****2).

**Table 2:** Univariate associations between demographics (Wave 1) and subsequent vaccination uptake between Wave 1 and Wave 2.

Demographics	% of the sample	Association with subsequent vaccination uptake (OR, 95% CI) (N = 505)
Gender		
Male	38%	1.00
Female	62%	1.10 (0.62–1.94)
Age group (years)		
18–34	85%	1.00
≥35	15%	3.24 (1.77–5.94)^a^
Marital status		
Single	83%	1.00
Married or formerly married	17%	2.71 (1.56–4.72)^a^
Occupation		
Student	67%	1.00
Employee	33%	2.68 (1.62–4.45)^a^
Education		
Secondary or below	14%	1.00
≥tertiary	76%	0.67 (0.36–1.23)
With chronic conditions^b^	6%	4.55 (2.01–10.30)^a^
Past vaccination uptake	37%	5.18 (2.84–9.45)^a^

a p<0.001.

b chronic conditions such as hypertension and diabetes.

### Correlations between different risk perception measures

Table 3 showed that all risk perception measures were strongly correlated (p<0.01). For each type (dimension) of risk perception measure, a composite score was generated by summing all the items within that particular dimension. The internal consistencies for these composite dimension scores of risk probability judgment, beliefs about risk and feeling at risk were moderately high to high (Cronbach's alpha ranging from 0.87 to 0.90). In addition to the correlations between each dimension's individual item scores with their respective composite score (rs ≥0.89), high correlations were also found between “expect to get flu” and “feel will get flu”: (r = 0.91), suggesting that collinearity exists between the two items and that they may measure the same aspect of risk perception. Collinearity was also suggested between the composite scores of beliefs about risk and feeling at risk (r = 0.87) but not between the composite scores of risk probability judgment and both beliefs about risk (r = 0.65) or feeling at risk (r = 0.64).

**Table 3:** Bivariate Spearman correlation coefficient matrix.

Variables	P1	P2	P3	P4	CP	B1	B2	CB	F1	F2	CF	FVU
Risk probability judgment												
P1: 2-point verbal scale	1											
P2: 6-point verbal scale	0.51	1										
P3: 7-point verbal scale	0.48	0.72	1									
P4: Percentage scale	0.49	0.69	0.82	1								
CP (α = 0.87)	0.63	0.81	**0.89**	**0.96**	1							
Beliefs about risk												
B1: Sure will get flu	0.39	0.54	0.56	0.55	0.60	1						
B2: Expect to get flu	0.47	0.60	0.58	0.58	0.64	0.77	1					
CB (α = 0.88)	0.46	0.59	0.60	0.60	0.65	**0.93**	**0.94**	1				
Feeling at risk												
F1: Feel will get flu	0.47	0.58	0.57	0.55	0.62	0.75	**0.91**	**0.87**	1			
F2: Feel vulnerable to flu	0.45	0.57	0.56	0.57	0.61	0.71	0.82	0.81	0.81	1		
CF (α = 0.90)	0.49	0.60	0.59	0.58	0.64	0.76	**0.90**	**0.87**	**0.94**	**0.94**	1	
Vaccination uptake	0.13	0.29	0.29	0.28	0.28	0.27	0.31	0.29	030	0.30	0.31	1

All coefficients are statistically significant (p<0.01); Coefficients indicating collinearity are in bold font.

CP: Composite score of the four items of the risk probability judgment dimension; CB: Composite score of the two items of the beliefs about risk dimension; CF: Composite score of two items of the feeling at risk dimension.

### Relationships of different risk perception measures with vaccination uptake

Correlation coefficients of different risk perception measures with vaccination uptake were also shown in Table 3. Among all the items for measuring risk probability judgment, the 2-point scale had the weakest correlation with vaccination uptake (r = 0.13) while other scales seemed to have comparable correlations with vaccination uptake (r = 0.28/0.29). Items for measuring beliefs about risk and feeling at risk seemed to have slightly stronger correlation with vaccination uptake (rs ranged between 0.27 and 0.31) than items for measuring risk probability judgment. The composite score of the three dimensions of risk perception did not show to have stronger correlations with vaccination uptake (rs ranged between 0.28 and 0.31) than individual items within each dimension.

Subsequently, multiple regression analyses were conducted to assess the relative strengths of the four individual risk probability judgment items in predicting vaccination uptake. A total of six logistic regression models were conducted to regress vaccination uptake on any pair of the four probability judgment scales. All regression models were adjusted by significant demographics including age, marital status, occupation, chronic condition and past influenza vaccination, indentified in univariate analyses (Table 4). The results showed that the 7-point scale was significant and stronger in predicting vaccination uptake when paired with each of the other three scales (β ranging from 0.28–0.41, all p<0.05), while the 6-point scale was significant and stronger in predicting vaccination when paired with the 2-point scale (β = 0.44, p<0.001) or the percentage scale (β = 0.25, p<0.05) but not when paired with the 7-points cale. The percentage scale was only significant and stronger in predicting vaccination uptake when paired with the 2-point scale (β = 0.40, p<0.001). Finally, the 2-point scale was no longer a significant predictor and explained no additional variance in vaccination when paired with each of the other three scales (all ps>0.05). A post hoc analysis that included all four individual risk probability judgment items in a single logistic regression model found that none of these items were significant in predicting vaccination uptake but the standardized regression coefficient for the 7-point scale remained the largest compared with those for the other three scales (Model 7 in Table 4).

**Table 4:** Comparison of different individual items of risk probability judgment dimension (Wave 1) in predicting subsequent vaccination uptake (Wave 2).

	Association with subsequent vaccination uptake (N = 505)						
	Model 1	Model 2	Model 3	Model 4	Model 5	Model 6	Model 7
Risk probability judgment							
2-point verbal scale	−0.01	−0.01	−0.03	–	–	–	−0.05
6-point verbal scale	0.44^c^	–	–	0.19	0.25^a^	–	0.17
7-point verbal scale	–	0.41^c^	–	0.27^a^	–	0.28^a^	0.20
Percentage scale	–	–	0.40^c^	–	0.21	0.16	0.12
Age	0.06	0.06	0.10	0.06	0.08	0.07	0.07
Marital status	−0.02	−0.01	−0.03	−0.01	−0.02	−0.02	−0.01
Occupation	0.10	0.09	0.09	0.09	0.08	0.08	0.08
Chronic condition	0.10	0.10	0.10	0.10	0.10	0.10	0.10
Past flu vaccination	0.26^c^	0.27^c^	0.29^c^	0.25^c^	0.26^c^	0.27^c^	0.26^c^

a p<0.05, ^c^ p<0.001.

All data in the table were standardized regression coefficients.

Similar logistic regression models were conducted to assess the relative importance of different risk perception dimensions (risk probability judgment, beliefs about risk and feeling at risk) in predicting vaccination uptake. To minimize the influence of multicollinearity, the composite score of each risk perception dimension rather than the individual item score was used in the logistic regression model. The risk probability judgment was significant and a stronger predictor of vaccination uptake (β = 0.25, p = 0.029) when paired with beliefs about risk (β = 0.21, p = 0.052) (Model 1 in Table 5); when paired with feeling at risk, risk probability judgment remained a significant but weaker predictor for vaccination uptake (β = 0.23, p = 0.034) than was feeling at risk (β = 0.25, p = 0.021) (Model 2 in Table 5). Both beliefs about risk (β = 0.14, p = 0.321) and feeling at risk (β = 0.28, p = 0.067) became non-significant in predicting vaccination uptake when they were paired in the regression model (Model 3 in Table 5). These results indicate that probability judgment explained additional variance in vaccination uptake that could not be explained by beliefs about risk; probability judgment and feeling at risk jointly explained additional variance in vaccination uptake that was not explained by each separately; and finally, beliefs about risk and feeling at risk explained mostly the same variance in vaccination uptake. None of the three risk perception dimensions were significant in predicting vaccination uptake when they were entered into the same logistic regression model but the strength of prediction for probability judgment and feeling at risk was identical (β = 0.22) and stronger than beliefs about risk (β = 0.04) (Model 4 in Table 5). The absolute value of Log Likelihood of Model 2 (−LL = 132.75) was the smallest compared with that of Model 1 and Model 3, suggesting that Model 2 explained more variances in vaccination uptake compared with the other two models.

**Table 5:** Comparison of three risk perception dimensions in predicting subsequent vaccination uptake.

Independent variables	Association with subsequent vaccination uptake (N = 505)			
	Model 1 (β)	Model 2 (β)	Model 3(β)	Model 4 (β)
Probability judgment	0.25^a^	0.23^a^	–	0.22
Beliefs about risk	0.21	–	0.14	0.04
Feeling at risk	–	0.25^b^	0.28	0.22
Age	0.08	0.09	0.09	0.09
Marital status	−0.02	−0.02	−0.03	−0.02
Occupation	0.07	0.07	0.09	0.07
Chronic condition	0.11	0.10	0.11	0.10
Past flu vaccination	0.25^c^	0.25^c^	0.27^c^	0.25^c^
−Log Likelihood (−LL)	133.64	132.75	134.51	132.72

a p<0.05, ^b^ p<0.01, ^c^ p<0.01; the three risk perception dimensions were indexed by their respective composite score; all numbers showed in the table represent standardized regression coefficients except for the last row showing the −Log Likelihood of each model.

## Discussion

Using the best measures for risk perception is critical for improving assessment of the associations between risk perceptions and influenza vaccination to inform public health interventions on improving vaccination uptake. This prospective study evaluated relative effectiveness of different risk perception measures in predicting subsequent influenza vaccination uptake. After adjusting for some of the methodological problems of measuring risk perception in existing studies [20], [21], [30], this study gave a clearer indication of the predictive power of different risk perception measures regarding subsequent influenza vaccination uptake. The study sample was generally healthy. Although around 6% of the respondents reported having a chronic condition, the effect of which on vaccination uptake was adjusted for when examining the associations between risk perceptions measures and vaccination uptake. Therefore, respondents' vaccination decision-making was more likely to be volitional. This results in a “cleaner” assessment of the influences of influenza risk perception on vaccination decision-making.

Around 11% of the respondents reported having had received seasonal influenza vaccine between Wave 1 and Wave 2, suggesting a low rate of influenza vaccination uptake among Hong Kong respondents and slightly lower than that reported in similar studies conducted elsewhere [23], [31]. Seasonal influenza vaccination is an annually-promoted procedure. Consistent with previous studies, this study showed that past vaccination remained the strongest predictor of subsequent vaccination [32]. Although some demographic factors include age, marital status, occupation and whether presenting chronic conditions or not were significantly associated with vaccination uptake in the univariate analyses, the effects of these factors on vaccination uptake became insignificant after perceptions of influenza risk were included in the multivariate regression models. This suggests that these demographic variables affect vaccination uptake through their effects on perceptions of disease risks, consistent with the health belief model [16]. However, gender was not significant associated with subsequent vaccination uptake against seasonal influenza in this Chinese sample which is inconsistent with a recent Western study [23].

Among all the different types of risk perception assessment, feeling at risk (vulnerability) was the best predictor for subsequent vaccination uptake while risk probability judgment was better in predicting subsequent vaccination uptake than beliefs about risk. This results were consistent with that of Weinstein and others' study though their study did not compare the predictions of feeling at risk and beliefs about risk in the same group of sample [23]. Feeling at risk, which considered to reflect perceived vulnerability to influenza-related harm, capture the emotional dimension of risk perception and thereby predict action uptake better than solely-cognitive probability estimates [33]–[35]. In our sample, beliefs about risk, though weaker in predicting vaccination uptake, seemed to predict the same aspect of vaccination uptake as feeling at risk. The correlation matrix also showed high correlations between the item for measuring beliefs about risk (e.g., expect to get the flu) and the item for measuring feeling at risk (e.g., feel will get the flu), and between the composite score of the beliefs about risk and the feeling at risk dimensions. This suggests that these two items are measuring a similar construct in this Chinese population. It is possible that although the slight wording differences between items measuring beliefs about risk and feeling at risk which are semantically-differentiated by English speaking people, may not be equally differentiated by Chinese or other language groups. This may be due to translation inadequacy or semantic limitation of the language.

Of the four measures of risk probability judgment, the 7-point verbal scale was the strongest predictor for vaccination uptake followed by the 6-point verbal scale while the 2-point verbal scale was the poorest predictor of vaccination uptake. This is consistent with Weinstein and others' study [23]. Other studies also suggest that a seven-point verbal-anchored scale is the best measure of probability magnitude [36]. Although the percentage scale was better in predicting vaccination uptake than the 2-point scale in our sample, previous studies [23], [37], [38] have showed that people, particularly those with low economic status usually have difficulties in understanding percentage information. Therefore, caution is recommended when using this item for assessing risk perception.

While multiple item measures of constructs theoretically reduce measurement error [29] and thereby improve prediction, our study found no great advantage in using composite over single-item scores for predicting vaccination uptake. Future studies could consider using a single item instead of multiple items for assessing these risk perception measures when designing questionnaires to reduce assessment load.

Study limitations include first, a full response rate (30%) which may reflect many student participants graduating between Waves 1 and 2 and therefore excluded from the university intranet. This was suggested by the slightly younger of the respondents who were lost to follow in Wave 2. However, other demographic differences were not found between respondents who completed both waves of the survey and those who were lost to follow in the second wave, suggesting that the influence of non-response bias is likely small. Second, study participants were university students, staff or faculty, mostly well-educated compared to the general population. Therefore, caution is needed when extrapolating the findings of this study to the general population of Hong Kong, even though education level was not a significant predictor of vaccination uptake. Finally, our examination on the associations between baseline risk perception measures and subsequent vaccination uptake is inevitably influenced by the occurrence of the 2009 influenza A/H1N1 pandemic. We minimized this influence by excluding respondents who reported having had received A/H1N1 vaccine. Despite this, some respondents may have received the seasonal influenza vaccine to avoid A/H1N1 infection before A/H1N1 vaccine was available even though it was emphasized by the World Health Organization that A/H1N1 was a novel influenza virus [39].

In conclusion, this study showed that feeling at risk, a variable capturing affective-cognitive dimensions of risk best predicted subsequent vaccination uptake against seasonal influenza while risk probability judgment was better at predicting vaccination uptake than was beliefs about risk. In this Chinese sample beliefs about risk assessed a comparable dimension of risk perception as did the variable of feeling at risk. Among the four scales evaluated for assessing risk probability judgment, the 7-point scale offered the best predictive power while the 6-point scale was the next best and the 2-point scale the poorest predictor of subsequent vaccination uptake. Finally, the study found that composite scores offered little if any advantage over single-item measures in predicting seasonal influenza vaccine uptake.

## Supporting Information

Table S1 **Characteristics of the respondents who completed both Wave 1 and Wave 2 of the survey and who completed the Wave 1 but were lost to follow in Wave 2 of the survey.** ^a^ p-value for Chi-square test to compare the demographic differences between subjects who completed both waves of the survey and those who completed Wave 1 but lost to follow in Wave 2.
(DOCX)
