# Supplementary material for: Comparison of Different Risk Perception Measures in Predicting Seasonal Influenza Vaccination among Healthy Chinese Adults in Hong Kong: A Prospective Longitudinal Study
Source: PLoS One. 2013 Jul 19;8(7):e68019. doi: 10.1371/journal.pone.0068019 (PMC3716928; doi:10.1371/journal.pone.0068019)
Supplement: Table S1 — Characteristics of the respondents who completed both Wave 1 and Wave 2 of the survey and who completed the Wave 1 but were lost to follow in Wave 2 of the survey. a p-value for Chi-square test to compare the demographic differences between subjects who completed both waves of the survey and those who completed Wave 1 but lost to follow in Wave 2. (DOCX) [file pone.0068019.s001.docx]

Table S1. Characteristics of the respondents who completed both Wave 1 and Wave 2 of the survey and who completed the Wave 1 but were lost to follow in Wave 2 of the survey

| Characteristics | Wave 1 (n=1764) | Subjects who completed Wave1&2 (n=525) | Subjects who completed Wave 1 but not Wave 2 (n=1239) | Differences (p)^a^ |
| --- | --- | --- | --- | --- |
| Gender |  |  |  |  |
| Female | 61% | 60% | 62% | 0.479 |
| Male | 38% | 40% | 38% |  |
| Age group |  |  |  |  |
| 18-34 | 85% | 82% | 87% | 0.025 |
| ≥35 | 15% | 18% | 13% |  |
| Marital status |  |  |  |  |
| Single | 83% | 82% | 83% | 0.541 |
| Married or formerly married | 17% | 18% | 17% |  |
| Occupation |  |  |  |  |
| Student | 67% | 65% | 68% | 0.199 |
| employee | 33% | 45% | 32% |  |
| Education |  |  |  |  |
| ≤Secondary | 24% | 13% | 24% | 0.466 |
| Tertiary or above | 76% | 77% | 76% | 0.560 |

^a^ p-value for Chi-square test to compare the demographic differences between subjects who completed both waves of the survey and those who completed Wave 1 but lost to follow in Wave 2.
